# Bilateral Glaucoma as Possible Additional Feature for *PGAP3*-Associated Hyperphosphatasia

**DOI:** 10.1155/2024/3561555

**Published:** 2024-03-23

**Authors:** Osama Obaid, Reem Batawi, Heba Alqurashi, Thana Ewis, Ahmad A. Obaid

**Affiliations:** ^1^Department of Pediatrics, Maternity and Children Hospital, Makkah, Saudi Arabia; ^2^Department of Radiology, Maternity and Children Hospital, Makkah, Saudi Arabia; ^3^Department of Laboratory Medicine, Faculty of Applied Medical Sciences, Umm Al Qura University, Makkah, Saudi Arabia

## Abstract

Hyperphosphatasia with mental disorder (HPMRS) is a rare autosomal recessive disease caused by gene mutations in enzymes involved in the synthesis and remodeling of lipids. Seven-month-old boy diagnosed with bilateral glaucoma had a cleft palate, facial dysmorphism, hypertelorism, a broad nasal bridge, and large fleshy earlobes. A brain MRI scan also revealed brain abnormalities. The observed phenotype in a seven-month-old boy is in agreement with the phenotypic features of HPRMS type-4. Whole exome sequencing revealed a possible pathogenic variant of *PGAP3* in a homozygous state (c.320C > T, p.Ser107Leu) which supported the diagnosis of HPRMS type-4. We report an unusual presentation for HPMRS and suggest adding this syndrome to the list of differential diagnoses of syndromic congenital glaucoma.

## 1. Introduction

Congenital disorders of glycosylation are a heterogeneous group of metabolic and genetic disorders caused by either autosomal recessive or dominant gene mutation of enzymes which are involved in glycan synthesis and modification of pathways [1]. Glycosylphosphatidylinositol anchor deficiency is an emerging class of congenital disorders of glycosylation reflecting multiple phenotypic features including facial dysmorphism, reduced muscle tone, intellectual disability, epilepsy, and various anomalies [2, 3]. Hyperphoshphatasia with mental disorder (HPMRS) is referred to as Mabry syndrome, a rare autosomal recessive disease with a wide spectrum of phenotypic features including mental disability, seizures, increased alkaline phosphatase levels, brachytelephalangy, and coarse facial characteristics (wide nasal bridge, hypertelorism, tented narrow upper lip, and prolonged palpebral fissure) [4, 5]. There are six different subclasses of HPMRS, and mutation in these genes affects the remodeling (*PGAP2* and *PGAP3*) or synthesis (*PIGO*, *PIGW, PIGY,* and *PIGV*) of anchor proteins [6]. A mutation in post-GPI attachment to protein 3 (*PGAP3*) gene is linked with HPMRS type-4 that encodes a glycosylphosphatidylinositol (GPI)-specific phospholipase, which is responsible for the synthesis of GPI-anchored proteins (GPI-APs). Several lines of evidence have reported the association between compound heterozygous or homozygous mutation with HPRMS type-4 [6–9].

All previously reported cases of pathogenic variants in *PGAP3* showed global developmental delay, cleft palate, brain anomalies, and elevated alkaline phosphatase levels (OMIM# 615716).

Here, we present a new case of *PGAP3* associated with a novel eye malformation that may expand the phenotype of this syndrome.

## 2. Clinical Case Presentation

**Figure 1:**
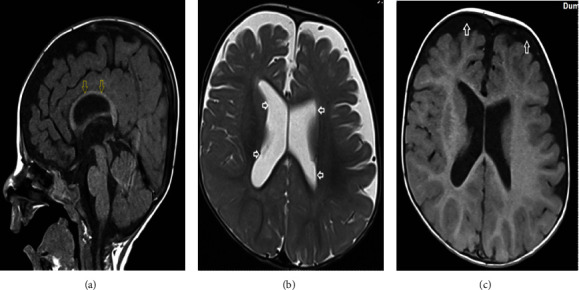
Brain MRI of a 10 months old boy in sagittal T1-WI (a) showing diffusely thin corpus callosum (yellow arrows), axial T2-WI (b) showing mildly dilated lateral ventricles (white horizontal arrows), and axial T1-WI (c) showing dilated subarachnoid spaces (white vertical arrows).

Seven-month-old boy born to consanguineous parents (first cousin), cesarean section due to maternal cause. A pediatric ophthalmologist diagnosed him with bilateral glaucoma and a cleft palate at the age of 3 months. The child was referred to a genetic clinic because of a delay in milestones and severe hypotonia for Pierre Robin sequence and coarse facial features. He has facial dysmorphism in the form of hypertelorism, a broad nasal bridge, large fleshy earlobes, poor growth, and microcephaly. MRI brain showed a thin corpus callosum with dilated lateral ventricles (Figure 1). A previous study by Abdel-Hamid et al. [6] reported abnormalities in the brain on an MRI scan for *PGAP3* mutation.

We performed a whole-exome sequence in a commercial lab after obtaining consent from parents due to the complex phenotype. This study revealed a possible pathogenic variant of *PGAP3* (NM_033419.5): c.320C > T (p.Ser107Leu), in a homozygous state that can be associated with HPRMS type-4. The exome sequencing study did not reveal any other variant related to congenital glaucoma and no other secondary or incidental findings. Retrospectively alkaline phosphatase levels were elevated which can provide support for this diagnosis, and parents declined carrier testing which is supported by various studies [6, 10]. The family was fully counseled and referred for peri-implantation genetic testing and antenatal testing to prevent the recurrence of this rare syndrome.

## 3. Discussion

We report a previously reported homozygous likely pathogenic variant in *PGAP3* associated with unusual clinical presentation of HPRMS4. Our patient presented at birth with bilateral primary congenital glaucoma corrected at the age of 3 months. Knaus et al. [8] reported our variant previously in one patient with severe global developmental delay and elevated alkaline phosphatase levels. Our patients with bilateral glaucoma had cleft palates, which is an extremely common congenital abnormality, as it was diagnosed in >60% of cases of HPMRS type-4 disease. Other well-known syndromic causes of glaucoma include Stickler syndrome, osteogenesis imperfecta, and Aicardi–Goutieres syndrome. In addition to the known syndromes, these findings always had a classic and clear phenotype.

Congenital glaucoma is predominantly found in small families, which causes difficulties in studying the genetics of this disease. Developed techniques, including molecular genetics technology and applications of whole-exome sequencing and next-generation sequencing (NGS), have a significant value for the study of cases of glaucoma, particularly in small families [11]. The elucidation of the pathogenesis of primary congenital glaucoma may be assisted by exploring causative genes, identifying candidate variants, and understanding the functional relevance of these genes and mutations. The second generation or NGS technology has the advantage of being able to analyze short read lengths. With developments in genetic sequencing technology, it may be possible to reach a final molecular diagnosis for all cases presented with congenital glaucoma.

## 4. Conclusion

We report an unusual presentation for HPMRS type 4 and suggest adding this syndrome to the list of differential diagnoses of syndromic congenital glaucoma.

## Data Availability

The data supporting the current study are available from the corresponding author upon request.
